# (Fe_0.2_Ni_0.8_)_0.96_S tubular spheres supported on Ni foam as an efficient bifunctional electrocatalyst for overall water splitting

**DOI:** 10.1038/s41598-018-27477-z

**Published:** 2018-06-21

**Authors:** Peiman Xu, Jingwei Li, Jiaxian Luo, Licheng Wei, Dawei Zhang, Dan Zhou, Weiming Xu, Dingsheng Yuan

**Affiliations:** School of Chemistry and Materials Science, Jinan University, Guangzhou, 510632 People’s Republic of China

## Abstract

Earth-abundant and efficient bifunctional electrocatalysts for both hydrogen evolution reaction (HER) and oxygen evolution reaction (OER) are highly significant for renewable energy systems. However, the performance of existing electrocatalysts is usually restricted by the low electroic conductivity and the limited amount of exposed active sites. In this work, (Fe_0.2_Ni_0.8_)_0.96_S tubular spheres supported on Ni foam have been prepared by a sulfuration of FeNi layered double hydroxide spheres grown on Ni foam. Benefiting from the unique tubular sphere architecture, the rich inner defects and the enhanced electron interactions between Fe, Ni and S, this electrocatalyst shows low overpotential of 48 mV for HER at 10 mA cm^−2^ in 1.0 mol L^−1^ KOH solution, which is one of the lowest value of non-previous electrocatalyts for HER in alkaline electrolyte. Furthermore, assembled this versatile electrode as an alkaline electrolyzer for overall water splitting, a current density of 10 mA cm^−2^ is achieved at a low cell voltage of 1.56 V, and reach up to 30 mA cm^−2^ only at an operating cell voltage of 1.65 V.

## Introduction

Hydrogen energy has been regarded as the most promising alternative to replace those conventional fossil fuels due to its renewability, sustainability, high calorific value and great energy conversion with zero CO_2_ emission^1–3^. Electrochemical water splitting including the cathodic hydrogen evolution reaction (HER) and the anodic oxygen evolution reaction (OER), which provides us with a feasible method for sustainable hydrogen production^4^. However, both the HER and OER are greatly limited by the high overpotential and low electrocatalytic efficiency. It is thus imperative to explore efficient electrocatalysts. At present, the most active electrocatalysts for HER and OER are Pt and RuO_2_/IrO_2_, respectively, but the wide-spread utilization of these catalysts is restricted by their high cost and scarcity. Therefore, enormous efforts have been made to design alternative electrocatalysts, such as transition-metal chalcogenides^5–7^, phosphides^8^, alloys^9^, nitrides^10^, carbides^11^ for HER and transition-metal oxides/hydroxides^12,13^, phosphates^14^, borates^15^ for OER. Nevertheless, most of these researches concentrate on the perfection of either HER or OER activity. In consideration of simplifying the fabrication procedures and decreasing the overall cost for water splitting system, the exploitation of efficient and low-cost bifunctional electrocatalysts for both HER and OER is highly attractive and desirable^16–21^.

Transition-metal layered double hydroxides (LDHs) have exhibited efficient performance for both HER and OER, while their electrocatalytic activity is still limited by the low electronic conductivity^22–24^. Interestingly, we have noticed that the enhanced electronic conductivity and electrocatalytic performance have been achieved after converting the LDHs to transition-metal sulfides (TMSs)^25–27^. Recently, it has been confirmed that the charged states of sulfur and metals in TMSs are similar to those in hydrogenase and its analogs, which can severally as the proton-acceptors and hydride-acceptors in the water splitting processes and thereby result in efficiently catalytic activity^25^. Also, the inner defect-rich structure of TMSs has been attracting great attention because it can expose more active sites, and thus lead to outstanding improvement of electrocatalytic performance^28^.

Besides, the nanostructure modification is an effective way to optimize the electrocatalytic activity^29,30^. Comparing to the solid structures of materials (nanosheets, nanoneedles, nanowires and so on), hollow micro/nanostructures have a kinetically favorable open structure, more exposed active sites and even a shortened ion diffusion length, and thereby possess high activity for HER and OER^31–33^. Guan *et al*.^32^ had fabricated CoS_2_ nanotube arrays on a carbon cloth as a bifunctional electrocatalyst for overall water splitting, achieving a current density of 10 mA cm^−2^ at a cell voltage of 1.67 V. Zhang *et al*.^30^ had reported the alkaline electrolyzer assembled by carbon paper/carbon tubes/cobalt-sulfide sheets electrode needed a cell voltage of 1.743 V to reach 10 mA cm^−2^. Moreover, Chao *et al*.^33^ had synthesized Co_9_S_8_ hollow microspheres exhibited efficiently electrocatalytic activity for OER, HER, and even the oxygen reduction reaction. In spite of these crucial advances have been done, the rational design and construction of TMSs electrocatalysts with uniquely hollow nanostructure and high performance for both HER and OER still needs more efforts.

Herein, we have prepared (Fe_0.2_Ni_0.8_)_0.96_S tubular spheres on Ni foam ((Fe_0.2_Ni_0.8_)_0.96_S TSs/Ni) *via* vulcanizing FeNi-LDH spheres grown on Ni foam (FeNi-LDH Ss/Ni). As expected, owing to the unique tubular sphere architecture to facilitate the release of gaseous products, the rich inner defects to expose more active sites, and the strong electron interactions between the Fe, Ni and S to improve the charge-transfer kinetics, the (Fe_0.2_Ni_0.8_)_0.96_S TSs/Ni possesses improved performance for both HER and OER in basic electrolytes, compared with the FeNi-LDH Ss/Ni. It just needs overpotentials of 48 mV for HER and 233 mV for OER to drive a current density of 10 mA cm^−2^. Especially, an alkaline electrolyzer assembled by the (Fe_0.2_Ni_0.8_)_0.96_S TSs/Ni electrode can be driven by a single 1.5 V AA battery, demonstrating greatly practical prospect and feasibility for water splitting.

## Experimental

### Materials

Fe(NO_3_)_3_·9H_2_O and Ni(NO_3_)_2_·6H_2_O were purchased from Tianjin Yongda Chemical. CO(NH_2_)_2_ (urea), NH_4_F, and CH_3_CSNH_2_ (thioacetamide) were obtained from Aladdin. Ni foam was acquired from Kunshan Electronic Limited Corporation. All chemicals were directly used as received without any purification.

### Synthesis of the FeNi-LDH Ss/Ni

The Ni foam (3 cm × 3 cm) was cleaned with 3.0 mol L^−1^ HCl solution and absolute ethanol with an ultrasound for 10 min each. 484.8 mg Fe(NO_3_)_3_·9H_2_O, 872.4 mg Ni(NO_3_)_2_·6H_2_O, 303.0 mg urea and 18.5 mg NH_4_F were dissolved in a solution comprised of 30 mL distilled water and 20 mL absolute ethanol under vigorous stirring for 10 min. Then, the above solution was transferred into an 80 mL Teflon-lined stainless-steel autoclave, in which contained a piece of cleaned Ni foam. The autoclave was heated at 120 °C for 6 h and allowed to cool to room temperature naturally. The product was taken out, washed with distilled water, and dried at 60 °C.

### Synthesis of (Fe_0.2_Ni_0.8_)_0.96_S TSs/Ni

Typically, 300.5 mg thioacetamide (TAA) was dissolved in a solution consisted of 40 mL distilled water and 10 mL absolute ethanol. The above solution was transferred into an 80 mL autoclave, containing a piece of as-prepared FeNi-LDH Ss/Ni. The autoclave was maintained at 140 °C for 4 h. After cooling down to room temperature, the product was taken out and severally washed with distilled water and absolute ethanol for four times. Finally, the (Fe_0.2_Ni_0.8_)_0.96_S TSs/Ni was obtained after drying at 60 °C overnight. The mass loading is 2.5 mg cm^−2^.

### Synthesis of Ni_3_S_2_/Ni and FeS/Ni

The Ni-based precursor was fabricated by the analogic step as mentioned above, whereas without introducing Fe(NO_3_)_3_·9H_2_O, and altering the amount of Ni(NO_3_)_2_·6H_2_O to 1.2 g, followed by the same sulfuration process. For FeS, the similar procedures without adding the Ni foam and replacing Ni(NO_3_)_2_·6H_2_O with Fe(NO_3_)_3_·9H_2_O (1.7 g) were employed. Then the FeS were bonded to a Ni foam to form FeS/Ni (see the XRD results in Fig. S1).

### Characterizations

The X-ray diffraction (XRD) analysis was recorded by a MSAL-XD2 X-ray diffractometer using Cu Kα radiation (λ = 1.5406 Å). The inductively coupled plasma optical emission spectrometer (ICP-OES) was tested by Perkin Elmer Optima 2000DV. The scanning electron microscopy (SEM) observations were performed on Philips SEM-XL30S microscope operated at 15 kV. Transmission electron microscopy (TEM), high-resolution transmission electron microscope (HRTEM) and energy dispersive X-ray spectroscopy (EDS) were characterized using a JEOL JEM-2100F instrument at 200 kV. Nitrogen adsorption isotherms were recorded on a Micromeritics TriStar 3000 Analyzer at −196 °C. The Brunauer-Emmett-Teller (BET) surface area was determined by adsorption data. The X-ray photoelectron spectroscopy (XPS) measurements were carried out by using a model of ESCALab250 with an Alumina Ka (1486.6 eV) source.

### Electrochemical measurements

The electrochemical measurements were performed in a conventional three-electrode setup controlled by a CHI 660D electrochemical workstation (CH Instruments, China). The as-prepared samples, Hg/HgO electrode and platinum foil were used as the working, reference, and counter electrodes, respectively. Linear sweep voltammetry (LSV) was conducted with a scan rate of 1 mV s^−1^. All potentials in this work were converted to the reversible hydrogen electrode (RHE) according to the Nernst equation:

1 $${E}_{{\rm{RHE}}}={E}_{{\rm{Hg}}/{\rm{HgO}}}+(0.098+0.059\,{\rm{pH}})\,{\rm{V}}$$

Tafel plots were recorded *via* the Tafel equation:

2 $$\eta ={b}\,\mathrm{log}\,{j}+a$$

where *η* is the overpotential, *b* is the Tafel slope and *j* is the current density. Electrochemical impedance spectroscopy (EIS) experiments were performed in the frequency range from 10^5^ to 0.01 Hz with an amplitude potential of 5 mV. Chronoamperometry tests were implemented at certain potentials. The double layer capacitance (*C*_dl_) is proportional to the electrochemical surface area (ECSA), tested by cyclic voltammograms (CV) cycles with scanning rates of 4, 6, 8, 10, 12 and 14 mV s^−1^. The linear slope of capacitive currents versus scan rates is equal to 2*C*_dl_^34^.

## Results and Discussion

### Structure and morphology of (Fe_0.2_Ni_0.8_)_0.96_S TSs/Ni

**Figure 1:**
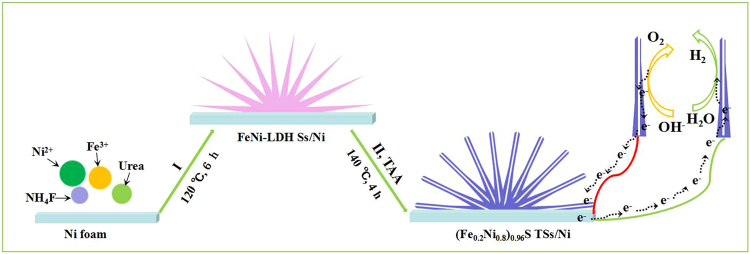
Schematic illustration of the synthesis of (Fe_0.2_Ni_0.8_)_0.96_S TSs/Ni.

**Figure 2:**
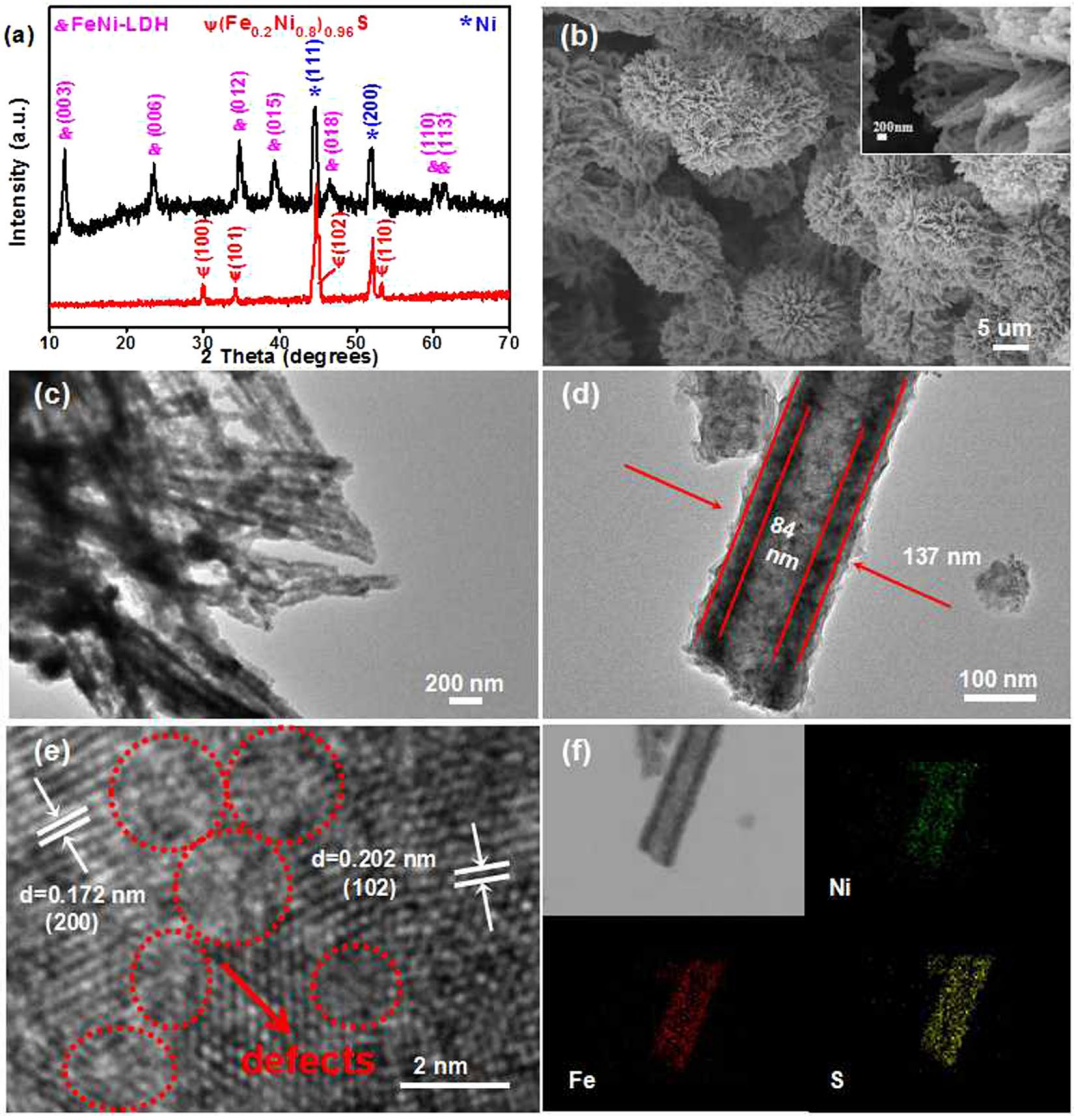
The characterizations of (Fe_0.2_Ni_0.8_)_0.96_S TSs/Ni. (**a**) XRD pattern; (**b**) SEM image; (**c** and **d**) TEM images; (**e**) HRTEM image and (**f**) elemental distribution mapping.

Figure 1 illuminates that the (Fe_0.2_Ni_0.8_)_0.96_S TSs/Ni has been prepared *via* two-step hydrothermal treatments. In step I, the FeNi-LDH Ss/Ni was prepared by the reactions between the metals ions and products released by the hydrolysis after heating at 120 °C for 6 h. The XRD pattern in Fig. 2a indicates the diffraction peaks at 11.4^ο^, 23.0^ο^, 34.4^ο^, 39.0^ο^, 46.0^ο^, 59.9^ο^, 61.3^ο^, corresponding to the (003), (006), (012), (015), (110) and (113) planes of rhombohedral Ni_0.75_Fe_0.25_(CO_3_)_0.125_(OH)_2_·0.38H_2_O (JCPDS no. 40–0215), respectively, matching well with the typical profile of LDH materials, and the other two peaks at 44.5^ο^ and 51.8^ο^ are indexed as the (111) and (200) planes of Ni foam (JCPDS no. 65–2865). In step II, the as-prepared FeNi-LDH Ss/Ni underwent a conversion *via* a sulfur treatment at 140 °C for 4 h, using TAA as the sulfur source to synthesize (Fe_0.2_Ni_0.8_)_0.96_S TSs/Ni. It can be seen that the diffraction peaks of FeNi-LDH are disappeared and the emerging peaks at 30.1°, 34.4°, 44.8° and 53.2° are attributed to the (100), (101), (102) and (110) planes of the hexagonal (Fe_0.2_Ni_0.8_)_0.96_S (JCPDS no. 50–1790). ICP-OES results indicate that the atomic ratio of Fe and Ni is about 0.2: 0.8 in Table S1, further confirming the successful formation of (Fe_0.2_Ni_0.8_)_0.96_S. Moreover, the EDS spectrum suggests the coexistence of Fe, Ni, and S, while the Cu elemental comes from the copper mesh (Fig. S2).

The SEM image in Fig. S3a reveals that the FeNi-LDH spheres with a diameter of about 10 μm are uniformly grown on the surface of Ni foam. Figure S3b further shows that these spheres consist of the nanoneedles. After vulcanization, the (Fe_0.2_Ni_0.8_)_0.96_S TSs/Ni retains the sphere characteristics of FeNi-LDH Ss/Ni, except for the enhanced surface roughness in Fig. 2b. As determined by N_2_ sorption measurement (Fig. S4a), the (Fe_0.2_Ni_0.8_)_0.96_S TSs/Ni exhibits a BET surface area of 73 m^2^ g^−1^, which is larger than that of FeNi-LDH Ss/Ni (53 m^2^ g^−1^). And the pore size distribution curve illustrates that both the mesopores and macropores exist in the (Fe_0.2_Ni_0.8_)_0.96_S TSs/Ni (Fig. S4b). The internal structure of (Fe_0.2_Ni_0.8_)_0.96_S TSs/Ni was further elucidated by TEM. Figure 2c and d show the (Fe_0.2_Ni_0.8_)_0.96_S spheres are comprised of the nanotubes with a pore diameter of 84 nm. Notably, the formation of (Fe_0.2_Ni_0.8_)_0.96_S tubular spheres can be explained by the Kirkendall diffusion effect^35–37^. The specific illustration is described as follows: when the solution is heated at 140 °C, it will release the S^2−^ ions from TAA. The S^2−^ will react with Fe and Ni ions to form a thin layer of (Fe_0.2_Ni_0.8_)_0.96_S on the surface of FeNi-LDH (Fig. S5). This thin layer can be a physical barrier to impede the direct reaction of the internal FeNi-LDH and the external S^2−^ ions. Then, the outward diffusion rate of the internal Fe and Ni ions is faster than the inward diffusion rate of external S^2−^ ions, resulting in the formation of tubular sphere structure of (Fe_0.2_Ni_0.8_)_0.96_S^38,39^. In addition, the HRTEM image shows lattice fringe spacing of 0.172 and 0.202 nm in Fig. 2e, which corresponds to the (110) and (102) planes of (Fe_0.2_Ni_0.8_)_0.96_S, respectively. Significantly, compared with the FeNi-LDH Ss/Ni (Fig. S3c), abundant defects can be observed in the HRTEM image of (Fe_0.2_Ni_0.8_)_0.96_S, which may provide more enrich active sites^40^, and further improve the electrocatalytic activity. The elemental mappings in Fig. 2f further indicate the homogeneous distribution of Ni, Fe and S in (Fe_0.2_Ni_0.8_)_0.96_S TSs/Ni.

XPS analysis was employed to investigate the surface chemical states on the (Fe_0.2_Ni_0.8_)_0.96_S TSs. As shown in Fig. S6a, the Fe 2p region displays the Fe 2p_3/2_ and Fe 2p_1/2_ peaks at 713.5 eV and 723.5 eV, respectively, indicating the Fe^3+^ oxidation states of (Fe_0.2_Ni_0.8_)_0.96_S TSs, while another peak at 707.6 eV is assigned to Fe-S bond^26,41,42^. The Ni 2p XPS spectrum in Fig. S6b shows that the peaks at 856.1 (Ni 2p_3/2_) and 874.1 eV (Ni 2p_1/2_), as well as their satellite peaks, are assigned to Ni^2+^, and the peak at 857.3 eV is attributed to Ni^3+ ^^43^. It is worth noting that the Fe 2p and Ni 2p peaks of (Fe_0.2_Ni_0.8_)_0.96_S TSs show a little positive shift after vulcanizing the FeNi-LDH Ss (Fig. S6a and b). Meanwhile, the spectrum of S 2p in Fig. S6c reveals the negatively shift peak at 161.6 eV with respect to the S and the peak at 162.8 eV is corresponding to the metal sulfide bonds^18,26,44^. The positive shift of Fe 2p and Ni 2p binding energy and negative shift of S 2p binding energy for (Fe_0.2_Ni_0.8_)_0.96_S TSs are mainly attributed to the influence of electron transfer between FeNi LDH Ss and (Fe_0.2_Ni_0.8_)_0.96_S TSs, suggesting the enhanced electron transfer from Fe, Ni to S^26,42,45^. Correspondingly, the relative interaction between Fe, Ni and S are enhanced after the sulfuration, which is beneficial to the charge transfer in the electrocatalysis process and hence favors the electrocatalytic activity.

### HER electrocatalytic activity

**Figure 3:**
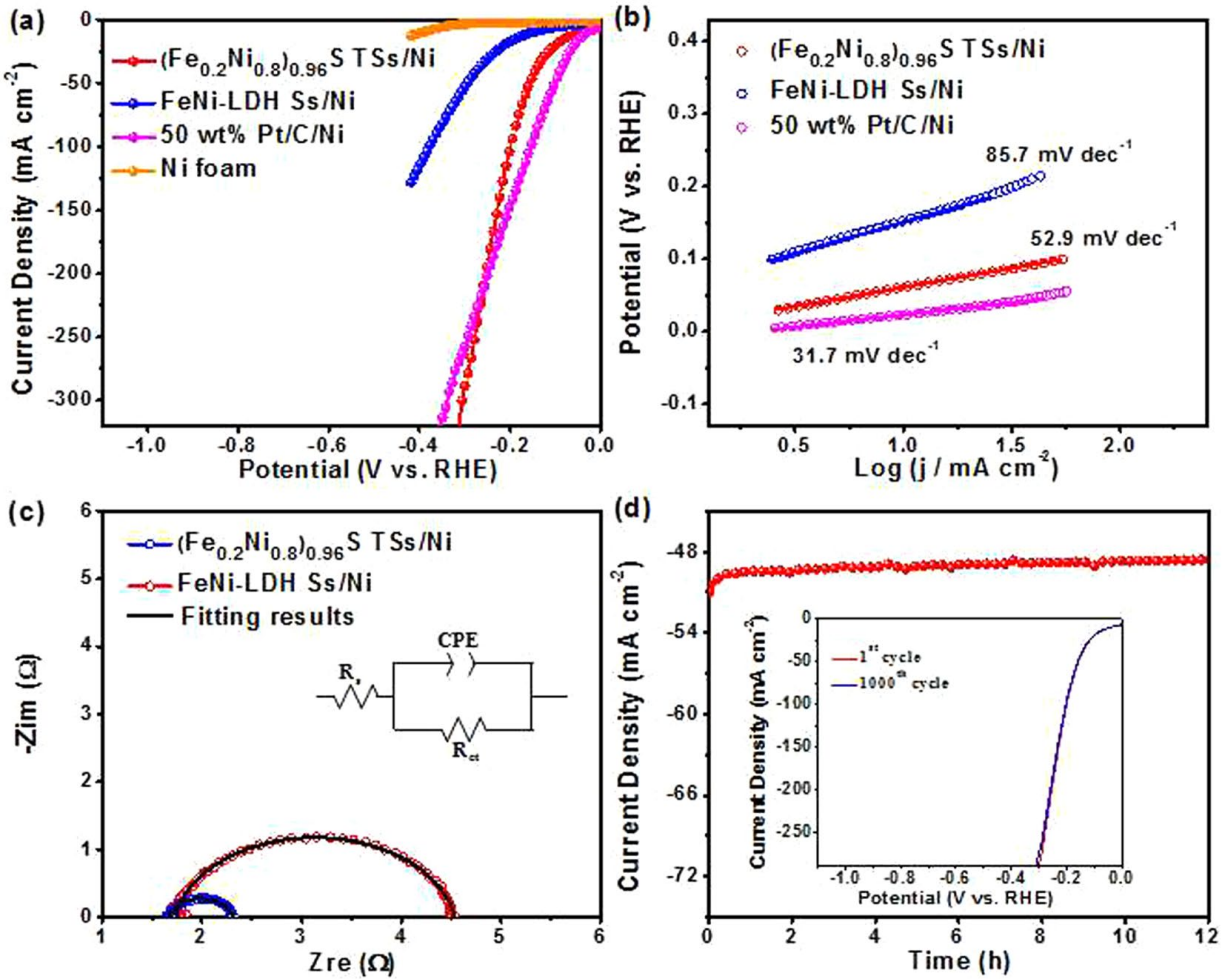
(**a**) Polarization curves; (**b**) Tafel plots; (**c**) Nyquist plots of (Fe_0.2_Ni_0.8_)_0.96_S TSs/Ni, FeNi-LDH Ss/Ni measured at overpotential of 330 mV; (**d**) Chronoamperometric measurement of the (Fe_0.2_Ni_0.8_)_0.96_S TSs/Ni at an overpotential of 154 mV. Inset is the polarization curves before and after 1000 cycles.

Electrocatalytic activity of the (Fe_0.2_Ni_0.8_)_0.96_S TSs/Ni for HER was assessed by LSV curves in a standard three-electrode system. For comparison, the FeNi-LDH Ss/Ni, 50 wt% Pt/C loading on Ni foam (50 wt% Pt/C/Ni^18^, the loading of 2.5 mg cm^−2^) and the bare Ni foam were also tested. Figure 3a shows the polarization curves of the above electrodes for HER at a scan rate of 1 mV s^−1^. Unquestionably, 50 wt% Pt/C/Ni exhibits the best performance with an onset potential close to zero, while bare Ni foam shows almost no HER performance. The as-prepared (Fe_0.2_Ni_0.8_)_0.96_S TSs/Ni exhibits excellent HER activity with sharp increase of current density, which requires low overpotentials of 48 mV and 198 mV to deliver the current density of 10 mA cm^−2^ and 100 mA cm^−2^ (*ƞ*_10_ = 48 mV, *ƞ*_100_ = 198 mV), respectively, outperforming that of *ƞ*_10_ = 153 mV and *ƞ*_100_ = 380 mV for FeNi-LDH Ss/Ni. This result indicates the enhanced HER activity of (Fe_0.2_Ni_0.8_)_0.96_S TSs/Ni after the sulfuration. Such excellent HER activity of (Fe_0.2_Ni_0.8_)_0.96_S TSs/Ni is also superior to the reported noble-metal-free materials listed in Table S2.

The corresponding Tafel plots were carried out to estimate the catalytic kinetics for HER. Figure 3b shows the Tafel slope of 31.7 mV dec^−1^ for 50 wt% Pt/C/Ni is well consistent with the previous report^18^. As expected, the Tafel slope of the (Fe_0.2_Ni_0.8_)_0.96_S TSs/Ni is 52.9 mV dec^−1^, much smaller than that of the FeNi-LDH Ss/Ni (85.7 mV dec^−1^), implying more favorable kinetics on (Fe_0.2_Ni_0.8_)_0.96_S TSs/Ni. To further evaluate the charge-transfer kinetics, the EIS of (Fe_0.2_Ni_0.8_)_0.96_S TSs/Ni and FeNi-LDH Ss/Ni was tested. As displayed in Fig. 3c, the charge transfer resistance (*R*_ct_) of 0.65 Ω for (Fe_0.2_Ni_0.8_)_0.96_S TSs/Ni is obviously lower than that of the FeNi-LDH Ss/Ni (2.79 Ω), suggesting better charge-transfer kinetics in the HER electrochemical processes. The lower charge-transfer resistance of (Fe_0.2_Ni_0.8_)_0.96_S TSs/Ni comparing to FeNi-LDH Ss/Ni probably arises from its unique tubular sphere architecture with a shortened ion diffusion length and the optimization of electron interactions between Fe, Ni and S after the sulfuration.

Apart from the electrocatalytic activity, the stability is another key evaluating parameter to assess practical value of electrocatalysts. Therefore, the stability of (Fe_0.2_Ni_0.8_)_0.96_S TSs/Ni was further measured by the chronoamperometric method. As shown in Fig. 3d, a stable current response suggests the (Fe_0.2_Ni_0.8_)_0.96_S TSs/Ni maintains the electrocatalytic activity after continuing hydrogen-release for 12 h. The polarization curve of (Fe_0.2_Ni_0.8_)_0.96_S TSs/Ni after 1000 cycles also reveals a negligible difference to the initial one (inset of Fig. 3d). Further SEM images, TEM image, N_2_ sorption isotherm and the corresponding pore-size distribution for (Fe_0.2_Ni_0.8_)_0.96_S TSs/Ni after stability measurements demonstrate that this electrocatalyst is still retained their tubular sphere structure with just a little aggregation (Fig. S7).

To get insight into the inherent electrocatalytic activity of (Fe_0.2_Ni_0.8_)_0.96_S TSs/Ni, the HER activities of Ni_3_S_2_/Ni and FeS/Ni were also investigated as shown in Fig. S8a. The (Fe_0.2_Ni_0.8_)_0.96_S TSs/Ni exhibits improved HER activity comparing to the Ni_3_S_2_/Ni and FeS/Ni. In addition, this electrocatalyst gives a smaller Tafel slope (52.9 mV dec^−1^) than Ni_3_S_2_/Ni (76.3 mV dec^−1^) and FeS/Ni (89.7 mV dec^−1^) (Fig. S8b). These results indicate that a significantly improved HER activity was achieved for (Fe_0.2_Ni_0.8_)_0.96_S TSs/Ni.

### OER electrocatalytic activity

**Figure 4:**
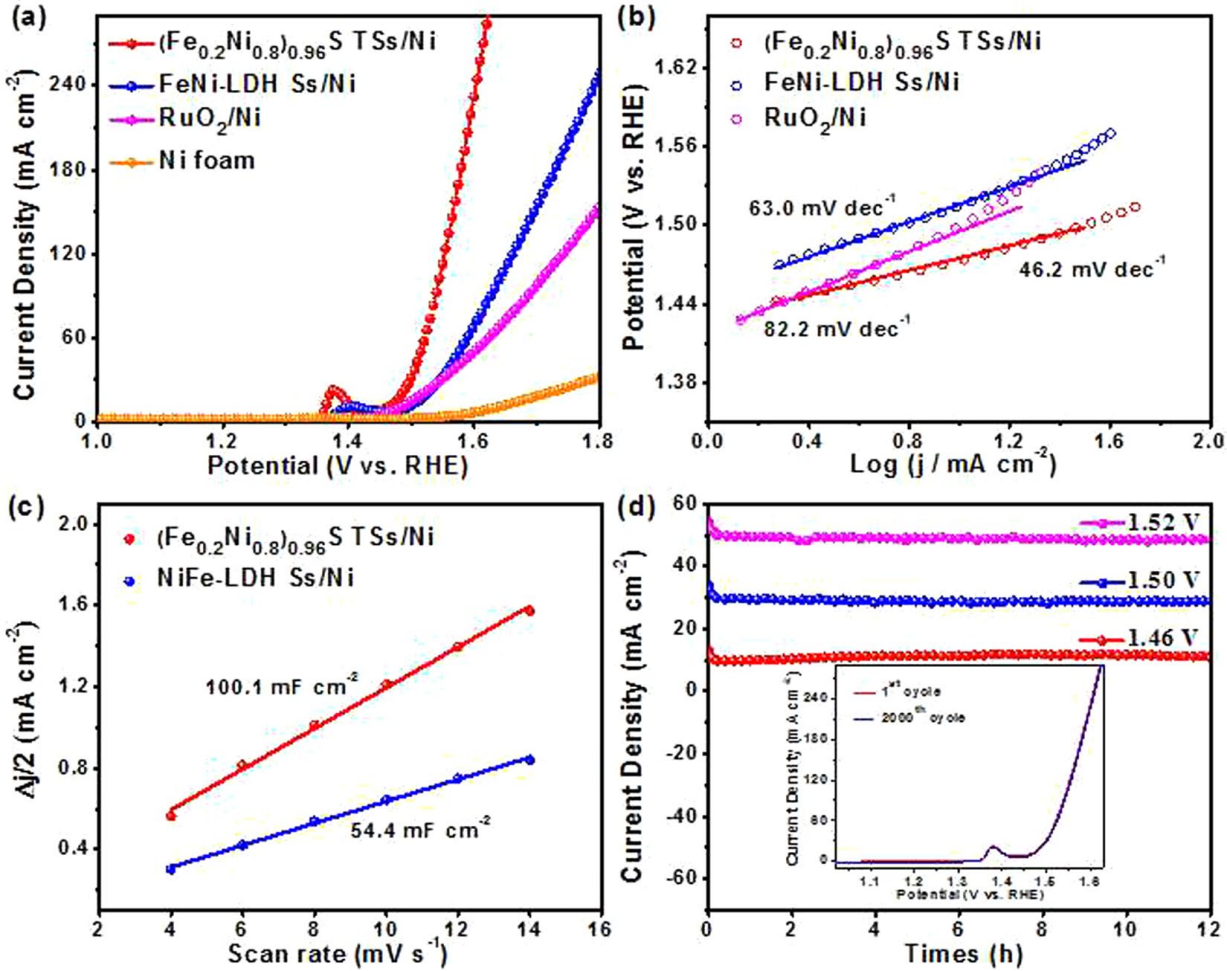
(**a**) Polarization curves; (**b**) Tafel plots; (**c**) Capacitive currents as a function of scan rate for (Fe_0.2_Ni_0.8_)_0.96_S TSs/Ni and FeNi-LDH Ss/Ni. (**d**) Chronoamperometric measurements of the (Fe_0.2_Ni_0.8_)_0.96_S TSs/Ni at different potentials. Inset is the polarization curves before and after 2000 cycles.

The electrocatalytic activity of (Fe_0.2_Ni_0.8_)_0.96_S TSs/Ni for OER in 1.0 mol L^−1^ KOH was further evaluated by LSV measurement at a scan rate of 1 mV s^−1^. Figure 4a exhibits the polarization curves for (Fe_0.2_Ni_0.8_)_0.96_S TSs/Ni, FeNi-LDH Ss/Ni, RuO_2_ loading on Ni foam (RuO_2_/Ni^18^, the loading of 2.5 mg cm^−2^) and the bare Ni foam. Interestingly, the anodic peak around 1.4 V vs RHE before OER can be observed for FeNi-LDH Ss/Ni, which is due to Fe and Ni species change to higher oxidation states^46,47^. However, the anodic peak of (Fe_0.2_Ni_0.8_)_0.96_S TSs/Ni shifted negatively to 1.38 V vs RHE, which may be attributed to the improved charge transfer. Specifically, the (Fe_0.2_Ni_0.8_)_0.96_S TSs/Ni exhibits a low *ƞ*_10_ of 233 mV and an *ƞ*_100_ of 310 mV for OER, superior to those of the FeNi-LDH Ss/Ni (*ƞ*_10_ = 263 mV), Ni foam (*ƞ*_10_ = 405 mV) and even the RuO_2_/Ni (*ƞ*_10_ = 253 mV). In addition, a smaller Tafel slope of (Fe_0.2_Ni_0.8_)_0.96_S TSs/Ni (46.2 mV dec^−1^) compares to those of FeNi-LDH Ss/Ni (63.0 mV dec^−1^) and RuO_2_/Ni (82.2 mV dec^−1^), suggesting a more favorable OER catalytic kinetics in Fig. 4b. The lowest overpotential and smallest Tafel slope highlight the excellent performance of (Fe_0.2_Ni_0.8_)_0.96_S TSs/Ni, which makes this uniquely tubular sphere as one of the best noble-metal-free electrocatalysts in Table S3. We further tested the OER activity for Ni_3_S_2_/Ni and FeS/Ni (Fig. S9a). The lowest overpotential of (Fe_0.2_Ni_0.8_)_0.96_S TSs/Ni to reach a current density of 100 mA cm^−2^ with respect to Ni_3_S_2_/Ni and FeS/Ni (Fig. S9b), indicating that the synergistic effect of Ni and Fe is an effector to improve the OER activity of (Fe_0.2_Ni_0.8_)_0.96_S TSs/Ni.

Besides, the electrochemically active surface area (ECSA) is an important parameter to understand the intrinsic activity of electrocatalysts, the double-layer capacitances (*C*_dl_) is herein employed in Fig. S10. As shown in Fig. 4c, the *C*_dl_ of 100.1 mF cm^−2^ for (Fe_0.2_Ni_0.8_)_0.96_S TSs/Ni is considerably larger than that of the FeNi-LDH Ss/Ni (54.4 mF cm^−2^), revealing the existence of enriched active sites on (Fe_0.2_Ni_0.8_)_0.96_S TSs/Ni. This is ascribed to the inner defect-rich crystal structure of (Fe_0.2_Ni_0.8_)_0.96_S TSs/Ni. The long-term stability of (Fe_0.2_Ni_0.8_)_0.96_S TSs/Ni for OER was tested at static potentials of 1.46, 1.50, and 1.52 V *vs*. RHE. Figure 4d shows an ignorable decrease of current density after continuing water oxidation for 12 h. The durability of (Fe_0.2_Ni_0.8_)_0.96_S TSs/Ni is also tested by using continuous CV sweeps in 1.0 M KOH at a scan rate of 100 mV s^−1^. The polarization curve reveals negligible degradation after 2000 cycles of CV scanning, confirming a satisfactory stability of (Fe_0.2_Ni_0.8_)_0.96_S TSs/Ni. Similar to HER, the SEM images, TEM image, N_2_ sorption isotherm and the corresponding pore-size distribution indicate the structure of (Fe_0.2_Ni_0.8_)_0.96_S TSs/Ni is insignificant change after OER durability testing, as displayed in Fig. S11.

To further investigate the nature of (Fe_0.2_Ni_0.8_)_0.96_S after OER performance, XPS is characterized. As shown in Fig. S12a, the sulfide peaks in the S 2p region is disappeared. The main peak at 531.7 eV in the O 1 s spectrum (Fig. S12b) indicates the oxidation of (Fe_0.2_Ni_0.8_)_0.96_S after OER tests. Moreover, the peaks located at 711.6 eV and 721.2 eV belonged to Fe-OOH bonds in the Fe 2p region are observed and the binding energy in the Ni 2p region shows a little positive shift, as shown in Fig. S12c and d, further reveling the Ni-Fe oxo/hydroxyl species were formed on the surface of the material. These results demonstrate that the superior OER electrocatalytic activity of (Fe_0.2_Ni_0.8_)_0.96_S could be attributable to the Ni-Fe oxo/hydroxyl species, consistent with the previous reports^26,48^.

### Overall water splitting

**Figure 5:**
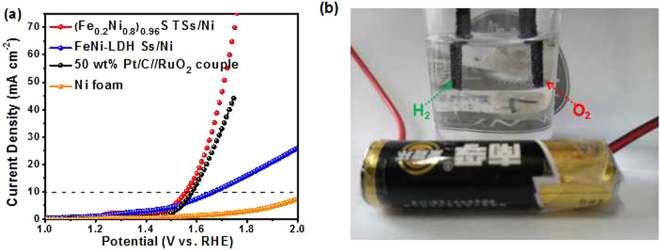
(**a**) Polarization curves; (**b**) photograph of (Fe_0.2_Ni_0.8_)_0.96_S TSs/Ni couple driven by a 1.5 V AA battery. The white bubbles of H_2_ and O_2_ can be observed in cathode and anode, respectively.

Inspired by the promising half-cell activity in HER and OER, the (Fe_0.2_Ni_0.8_)_0.96_S TSs/Ni was further served as anode and cathode in an electrolyzer for overall water splitting. As shown in Fig. 5a, this electrolyzer just requires low cell voltages of 1.56 V and 1.65 V to drive the current density of 10 mA cm^−2^ and 30 mA cm^−2^, respectively. Although the cell voltage of (Fe_0.2_Ni_0.8_)_0.96_S TSs/Ni couple to generate 10 mA cm^−2^ is larger than that of the Ni_x_Co_3-x_S_4_/Ni_3_S_2_/NF (1.53 V)^49^, it is superior to those of FeNi-LDH Ss/Ni (1.66 V), RuO_2_–50 wt% Pt/C couple (1.58 V), NiCo_2_S_4_@NiFe LDH/NF (1.60 V)^24^, Ni_0.7_Fe_0.3_S_2_/Ni (1.625 V)^26^, NiCo_2_S_4_/NF (1.68 V)^27^, CoS_2_ NTA/CC (1.60 V)^33^, FeSe_2_/NF (1.73 V)^50^, FeB_2_-NF (1.57 V)^51^, Zn_0.76_Co_0.24_S/CoS_2_/TM (1.66 V)^52^ and even most of the reported works exhibited in Table S4. Moreover, H_2_ and O_2_ with a predicted ratio of 2: 1 are obtained, and the amount of experimentally quantified gas is in good accordance with theoretically calculated gas, indicating that the (Fe_0.2_Ni_0.8_)_0.96_S TSs/Ni affords a Faradaic efficiency of ~100% for both HER and OER (Fig. S13). Such an electrocatalyst for overall water splitting can be also powered by a 1.5 V AA battery, as shown in Fig. 5b, demonstrating the considerable potential as an alkaline electrolyzer for practical applications.

The excellent electrocatalysis performance of (Fe_0.2_Ni_0.8_)_0.96_S TSs/Ni can be attributed to the following aspects: (1) the inner defect-rich crystal structure of (Fe_0.2_Ni_0.8_)_0.96_S TSs/Ni can expose more effectively active sites. Meanwhile, the outer unique tubular sphere architecture of (Fe_0.2_Ni_0.8_)_0.96_S TSs/Ni is beneficial to intimate contact between the material and electrolyte, and the release of gaseous products; (2) the change in composition between FeNi-LDH Ss/Ni and (Fe_0.2_Ni_0.8_)_0.96_S TSs/Ni leads to strong electron interactions between Fe, Ni and S, and further optimizes charge transfer; (3) the *in-situ* growth of (Fe_0.2_Ni_0.8_)_0.96_S TSs on Ni foam avoids the use of a binder, which can enhance the conductivity.

## Conclusions

In summary, (Fe_0.2_Ni_0.8_)_0.96_S TSs/Ni has been successfully synthesized *via* the hydrothermal sulfuration treatment of FeNi-LDH SAs/Ni. As expected, by taking advantage of the unique tubular sphere architecture, the rich inner defects and the enhanced electron interactions between Fe, Ni and S, the as-synthesized (Fe_0.2_Ni_0.8_)_0.96_S TSs/Ni possesses higher HER and OER performance with respect to FeNi-LDH Ss/Ni. Furthermore, the alkaline electrolyzer with (Fe_0.2_Ni_0.8_)_0.96_S TSs/Ni as the anode and cathode just needs cell voltages of 1.56 V and 1.65 V to achieve 10 mA cm^−2^ and 30 mA cm^−2^, respectively, suggesting the great value for the practical application. More importantly, this study will encourage new opportunities to design versatile electroactive materials with a uniquely hollow structure and high performance for water splitting, fuel cells, supercapacitors, and even the batteries.

## Electronic supplementary material

Supplementary Information
