# Supplementary material for: (Fe0.2Ni0.8)0.96S tubular spheres supported on Ni foam as an efficient bifunctional electrocatalyst for overall water splitting
Source: Sci Rep. 2018 Jun 21;8:9425. doi: 10.1038/s41598-018-27477-z (PMC6013453; doi:10.1038/s41598-018-27477-z)
Supplement: Supplementary file 1 — Supplementary Information [file 41598_2018_27477_MOESM1_ESM.doc]

**Supplementary Information**

**(Fe_0.2_Ni_0.8_)_0.96_S tubular spheres supported on Ni foam as an efficient bifunctional electrocatalyst for overall water splitting**

Peiman Xu^†^, Jingwei Li^†^, Jiaxian Luo, Licheng Wei, Dawei Zhang, Dan Zhou, Weiming Xu, Dingsheng Yuan*

School of Chemistry and Materials Science, Jinan University, Guangzhou 510632, People’s Republic of China

^†^ These authors contributed equally to this work.

* Corresponding Authors: [tydsh@jnu.edu.cn](mailto:tydsh@jnu.edu.cn)

1. **Faradic Efficiency**

Two-electrode water electrolysis was analyzed by chronopotentiometry measurement at a constant current of 10 mA cm^-2^ in 1.0 mol L^-1^ KOH solution. The oxygen and hydrogen bubbles were collected by a water splitting apparatus continuing for 180 min. The theoretical volume of O_2_ and H_2_ were calculated by the following equation:

*V_O2_* mL = Q C × 22.4 L mol^-1^ × 1000 / (F C mol^-1^ × 4)

*V_H2_* mL = Q C × 22.4 L mol^-1^ × 1000 / (F C mol^-1^ × 2)

where Q is the cumulative charge (C), F is the Faraday constant (C mol^-1^) ^1^.

1. **Figures**

**Figure S1.** XRD pattern of (a) Ni_3_S_2_/Ni; (b) FeS powders.

**Figure S2.** EDS spectrum of the (Fe_0.2_Ni_0.8_)_0.96_S TSs/Ni


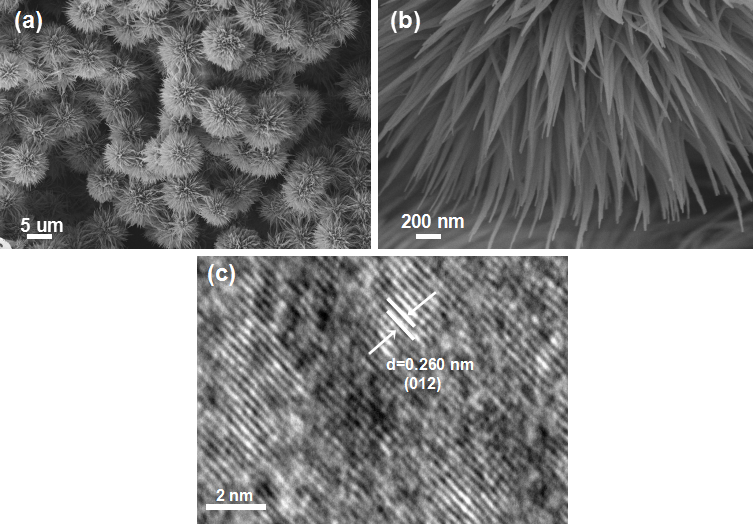


**Figure S3.** (a and b) SEM images; (c) HRTEM image of the FeNi-LDH Ss/Ni.

**Figure S4.** (a) N_2_ adsorption isotherms of the (Fe_0.2_Ni_0.8_)_0.96_S TSs/Ni and FeNi-LDH Ss/Ni. (b) the corresponding pore-size distributions of (Fe_0.2_Ni_0.8_)_0.96_S TSs/Ni.

**Figure S5.** The formation of (Fe_0.2_Ni_0.8_)_0.96_S nanotube.

**Figure S6.** XPS spectra of (a) Fe 2p and (b) Ni 2p for (Fe_0.2_Ni_0.8_)_0.96_S TSs/Ni and FeNi-LDH Ss/Ni; (c) S 2p XPS spectrum of (Fe_0.2_Ni_0.8_)_0.96_S TSs/Ni.


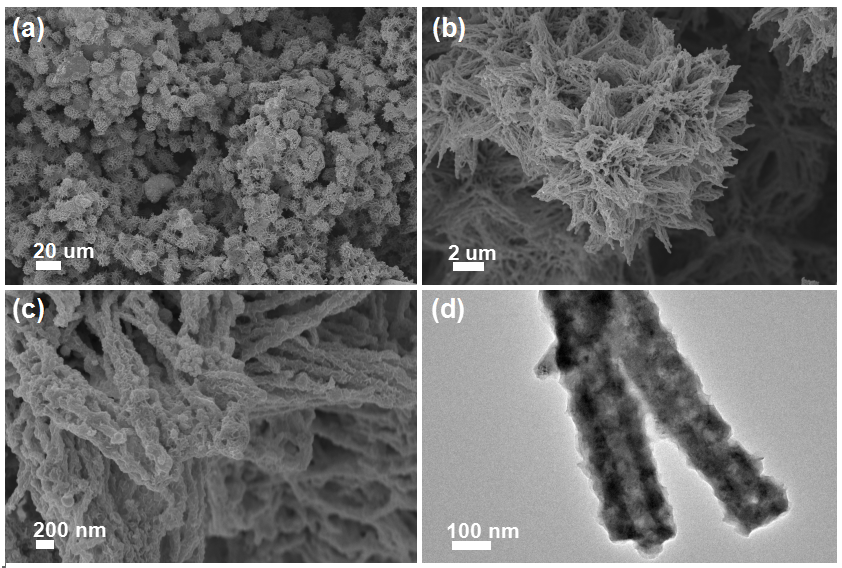


**Figure S7.** (a-c) SEM images; (d) TEM image; (e) N_2_ sorption isotherm and (f) the corresponding pore-size distribution of (Fe_0.2_Ni_0.8_)_0.96_S TSs/Ni after HER stability measurements.

**Figure S8.** (a) Polarization curves and (b) the corresponding Tafel plots of (Fe_0.2_Ni_0.8_)_0.96_S TSs/Ni, Ni_3_S_2_/Ni and FeS/Ni.

**Figure S9.** (a) Polarization curves and (b) the comparison of OER overpotential for (Fe_0.2_Ni_0.8_)_0.96_S TSs/Ni, Ni_3_S_2_/Ni and FeS/Ni at 100 mA cm^-2^.

**Figure S10.** Cyclic voltammograms of (a) (Fe_0.2_Ni_0.8_)_0.96_S TSs/Ni and (b) FeNi-LDH Ss/Ni in the region of 1.108 - 1.168 V *vs*. RHE.


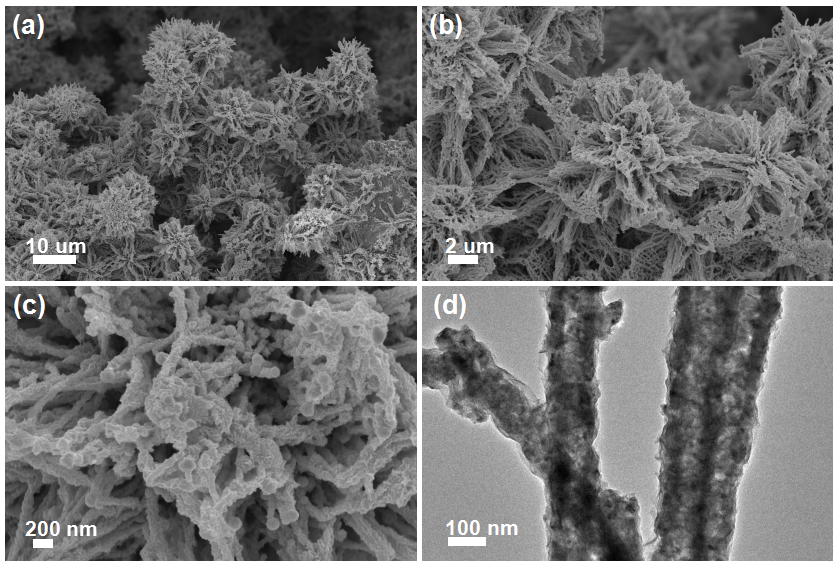


**Figure S11.** (a-c) SEM images; (d) TEM image; (e) N_2_ sorption isotherm and (f) the corresponding pore-size distribution of (Fe_0.2_Ni_0.8_)_0.96_S TSs/Ni after OER stability measurements.

**Figure S12.** XPS spectra of (a) S 2p; (b) O 1s; (c) Fe 2p; (b) Ni 2p for (Fe_0.2_Ni_0.8_)_0.96_S TSs/Ni after OER stability measurements.

**Figure S13.** Experimental and theoretical amounts of H_2_ and O_2_ by (Fe_0.2_Ni_0.8_)_0.96_S TSs/Ni at a current density of 10 mA cm^-2^ for 180 min.

**Table S1.** The actual ratio of element Fe and Ni for characterized (Fe_0.2_Ni_0.8_)_0.96_S TSs/Ni by ICP analysis.

| **Materials** | **Ions initial mole ratio of Fe and Ni** | **Fe   mg L^-1^** | **Ni   mg L^-1^** | **Percentage composition (Fe:Ni)** | **Actual ratio of elemental Fe and Ni** |
| --- | --- | --- | --- | --- | --- |
| (Fe_0.2_Ni_0.8_)_0.96_S TSs/Ni | 2.0 : 5.0 | 115.6 | 454.6 | 20.3% : 79.7% | 0.2 : 0.8 |

**Table S2**. Comparison of the HER performance of (Fe_0.2_Ni_0.8_)_0.96_S TSs/Ni with other

reported HER electrocatalysts in alkaline electrolyte.

| **Electrocatalysts** | ƞ@10mA cm^-2^ [mV] | ƞ@100mA cm^-2^ [mV] | Electrolyte  (KOH, mol L^-1^) | Ref. |
| --- | --- | --- | --- | --- |
| (Fe_0.2_Ni_0.8_)_0.96_S TSs/Ni | 48 | 198 | 1.0 | This work |
| NF-Ni_3_Se_2_/Ni | 203 | 279 | 1.0 | 2 |
| MoP/Ni_2_P/NF | 75 | 191 | 1.0 | 3 |
| Co-Mo_2_C-0.020 | 118 | 195 | 1.0 | 4 |
| MoSe_2_·Ni_0.85_Se@NF | 117 | 204 | 1.0 | 5 |
| NixCo_3x_S_4_/  Ni_3_S_2_/NF | 136 | 258 | 1.0 | 6 |
| MoS_2_-Ni_3_S_2_  HNRs/NF | 98 | 191 | 1.0 | 7 |
| Fe_0.45_Co_0.55_ composite films | 163 | 213 | 1.0 | 8 |

**Table S3**. Comparison of the OER performance of (Fe_0.2_Ni_0.8_)_0.96_S TSs/Ni with other

reported OER electrocatalysts in alkaline electrolyte.

| **Electrocatalysts** | ƞ@10mA cm^-2^ [mV] | ƞ@100mA cm^-2^ [mV] | Electrolyte  (KOH, mol L^-1^) | Ref. |
| --- | --- | --- | --- | --- |
| (Fe_0.2_Ni_0.8_)_0.96_S TSs/Ni | 233 | 310 | 1.0 | This work |
| NixCo_3x_S_4_/  Ni_3_S_2_/NF | 160 | 320 | 1.0 | 6 |
| MoS_2_-Ni_3_S_2_  HNRs/NF | 249 | 341 | 1.0 | 7 |
| Fe_0.4_Co_0.6_ composite films | 283 | 321 | 1.0 | 8 |
| NiCoP/CC | 242 | 330 | 1.0 | 9 |
| Fe-NiSe/NF | 233 | 275 | 1.0 | 10 |
| Co-S/Ti mesh | 361 | 440 | 1.0 | 11 |
| Ni_2.3%_-CoS_2_/CC | 270 | 370 | 1.0 | 12 |

**Table S4**. Comparison of electrocatalytic activity of (Fe_0.2_Ni_0.8_)_0.96_S TSs/Ni with recently reported bifunctional materials in alkaline electrolyte.

| **Electrocatalysts** | **HER** | **OER** | Cell voltage  for j=10 mA cm^-2^ | Electrolyte  (KOH, mol L^-1^) | Ref. |
| --- | --- | --- | --- | --- | --- |
|  | ƞ@10mA cm^-2^ [mV] | ƞ@10mA cm^-2^ [mV] |  |  |  |
| (Fe_0.2_Ni_0.8_)_0.96_S TSs/Ni | 48 | 233 | 1.56 | 1.0 | This work |
| MoS_2_-Ni_3_S_2_  HNRs/NF | 98 | 249 | 1.50 | 1.0 | 7 |
| NiCoP/CC | 62 | 242 | 1.52 | 1.0 | 8 |
| FeNi_3_N/NF | 75 | 202 | 1.62 | 1.0 | 13 |
| NiCo_2_S_4_  NW/NF | 210 | 260 | 1.63 | 1.0 | 14 |
| NSP-Co_3_FeN_x_ | 23 | 222 | 1.539 | 1.0 | 15 |
| Co_9_S_8_@NOSC | 320 | 340 | 1.60 | 1.0 | 16 |
| Ni/Mo_2_C-PC | 179 | 368 | 1.66 | 1.0 | 17 |
| CoP/GO-400 | 150 | 340 | 1.70 | 1.0 | 18 |
| CoSe_2_/CF | 95 | 297 | 1.63 | 1.0 | 19 |
| Co@CoO/NG-2 | 112 | 315 | 1.58 | 1.0 | 20 |
| CoNi_2_Se_4_ | 220 | 160 | 1.61 | 1.0 | 21 |

**References**

13. Zhang, B. *et al.* Iron-nickel nitride nanostructures in situ grown on surface-redox-etching nickel foam: efficient and ultrasustainable electrocatalysts for overall water splitting. *Chem. Mater.* **28**, 6934-6941 (2016).

14. Sivanantham, A., Ganesan, P. & Shanmugam, S. Hierarchical Ni_C_o_2_S4 nanowire arrays supported on Ni foam: an efficient and durable bifunctional electrocatalyst for oxygen and hydrogen evolution reactions*. Adv. Funct. Mate*r**.** 26, 4661-4672 (2016).

15. Wang, Y*. et al*. Porous cobalt-iron nitride nanowires as excellent bifunctional electrocatalysts for overall water splitting*. Chem. Commu*n**.** 52, 12614-12617 (2016).

1_6_. Huang, S*. et a*l. N-, O-, and S-tridoped carbon-encapsulated _C_o_9_S8 nanomaterials: efficient bifunctional electrocatalysts for overall water splitting*. Adv. Funct. Mate*r**.** 27, 1606585 (2017).

20. Zhang, S*. et a*l. N-Doped graphene-supported Co@CoO core-shell nanoparticles as high-performance bifunctional electrocatalysts for overall water splitting*. J. Mater. Chem.* **A** 4, 12046-12053 (2016).

21. Amin, B. G., Swesi, A. T., Masud, J. & Nath, M. Co_N_i2_S_e4 as an efficient bifunctional electrocatalyst for overall water splitting*. Chem. Commu*n**.** 53, 5412-5415 (2017).
